# The effects of shared medical appointment multidisciplinary interventions for non-organic feeding disorders in infants and young children during the self-feeding transition period

**DOI:** 10.3389/fped.2025.1595641

**Published:** 2025-05-16

**Authors:** Die Chen, Wen-Tao Peng, Xiao-Mei Liu, Fei Xiong, Yu Luo, Hong Luo, Meng-Yan Tang, Xin-Yu Guo, Xiao Fu, Qian Feng, Hong Chen

**Affiliations:** ^1^ Department of Nursing, West China Second University Hospital, Sichuan University, Chengdu, China; ^2^ Key Laboratory of Birth Defects and Related Diseases of Women and Children (Sichuan University), Ministry of Education, Chengdu, China; ^3^ Department of Child Health Care, West China Second University Hospital, Sichuan University, Chengdu, China

**Keywords:** feeding difficulties/disorder, non-organic, infants and young children, self-feeding transition period, shared medical appointment, intervention

## Abstract

**Objective:**

This study aimed to implement shared medical appointment multidisciplinary interventions for non-organic feeding disorders in infants and young children and evaluate their effects.

**Methods:**

A total of 52 children aged 6–24 months and their respective feeders were included in the study. Of them, 26 were classified into the intervention group, and 26 were classified into the control group. Routine child health care measures were applied to the control group. The child health care measures combined with shared medical appointment multidisciplinary interventions, including 3 collective interventions and 3 months of follow-up, were applied in the intervention group. Data concerning physical growth indicators, Montreal Children's Hospital Feeding Scale (MCH-FS) scores, Infant and Child Feeding Index (ICFI) scores, and Self-Rating Anxiety Scale (SAS) scores in the two groups were collected.

**Results:**

Due to insufficient participation in interventions, loss of follow-up, and withdrawal from the study, 46 cases were finally included in this study, with 23 cases in each group. The physical growth indicators in the intervention group were better than the control group, with the effects of time. The intervention group had lower MCH-FS score, higher ICFI score and lower SAS score.

**Conclusions:**

These results provide preliminary evidence of the effectiveness and feasibility of shared medical appointment multidisciplinary interventions, which help promote feeding and physical growth in infants and young children and provide a reference for improving management for feeding in infants and young children.

## 1 Introduction

Infants and young children get nutrition from feeding. The good nutritional status of infants and young children depends largely on good feeding. Despite rapid economic growth and improvements in healthcare services over the past few decades, malnutrition in children remains a major public health challenge in many countries. Broadly speaking, any behavior that does not conform to the eating ability and eating needs of infants and young children constitutes a feeding disorder. Feeding disorders are classified into organic and non-organic based on the presence or absence of underlying diseases. The World Health Organization defined non-organic feeding disorders as a child having weight loss or no weight gain under the premise of having no obvious physiological diseases (1).

According to the International Classification of Functioning, Disability and Health: Children and Youth version: ICF-CY, a multi-disciplinary expert consensus has put forward a unified diagnostic term for Feeding Difficulties - Pediatric Feeding Disorder (PFD) (2). PFD is defined as impaired oral intake that is not ageappropriate, and is associated with medical, nutritional, feeding skill, and/or psychosocial dysfunction. According to statistics, the incidence of feeding difficulties in infants and young children in China is 21.4% (3), as high as 68.4% in children who were born prematurely, and as high as 38.6% in children with low birthweight (4). Feeding disorders in infants and young children have become one of the main reasons for visits to child outpatient healthcare clinics. Studies by scholars from other countries have shown that the incidences of feeding difficulties in normal infants and young children vary from 25.0% to 35.0% (5).

The World Health Organization advocates exclusive breastfeeding for infants and young children for the first 6 months of life, recommends breastfeeding for the first 2 years of life, and defines the transition period from exclusive breastfeeding to family foods as 6–24 months of age, also referred as to “baby-led weaning (BLW)” (6, 7). This period is characterized by several transitions: from single to multiple types of food, from smaller to larger quantities of food and nutrient supplements, from more frequent meals to fewer meals, and from caregiver feeding to self-feeding. This transition period often involves changes in eating behaviors and has a high incidence of feeding disorders. Research by Chu and Sheng (8) showed that 81.48% of feeding disorder cases in children aged 0–3 years occurred in children aged 6–24 months. This was higher than the entire childhood.

Previous studies have demonstrated that feeding disorders in infants and young children should be solved by professional and multidisciplinary healthcare teams for systematic assessment, intervention, guidance, and management (9, 10). Healthcare professionals involving the improvement of feeding disorders are generally medical doctors, nurses, psychologists, nutritionists, speech-language pathologists, and occupational therapists (11). There is a general consensus on the intervention forms and measures for feeding disorders. However, few studies have conducted comprehensive evaluations, interventions, and follow-ups through multidisciplinary collaboration for non-organic feeding disorders in infants and young children who are in the self-feeding transition period. This study aimed to apply shared medical appointment multidisciplinary interventions for non-organic feeding disorders in infants and young children during the self-feeding transition period. The study evaluated its application effect in improving the signs of feeding disorders, eating behaviors, and feeding index scores of infants and young children, as well as changing the feeding behaviors of feeders.

## 2 Methods

### 2.1 Ethical approval

The study complied with the ethical standards of the Medical Research Ethics Committee and the Declaration of Helsinki. Ethical approval of this study was obtained from the Biomedical Ethics Committee of West China Second University Hospital, Sichuan University (Ethics approval document number: 2023060). Written informed consent was obtained from the children's feeders before the study. Participation in the study process is fully respectful of parents' opinions. Participants could decline or withdraw from the study at any time.

### 2.2 Study participants

This quasi-experimental study was conducted at a tertiary Grade A women's and children's hospital in Chengdu, Sichuan, China from March 2023 to June 2023. A total of 52 children aged 6–24 months and their respective feeders were included in the study. According to the outpatient registration numbers, participants were allocated to groups using a 1:1 ratio: children with odd registration numbers were assigned to the intervention group, while those with even registration numbers were assigned to the control group.

The sample size calculation in this study was based on the formula for “comparison of means between two independent samples”: n1 = n2 = 2 [(μα + μβ)^2^·*σ*^2^)/*δ*^2^], with *α* = 0.05 and *β* = 0.1. Using MCH-FS as the primary measurement indicator and adopting values from literature reports (*δ* = 6.31, *σ* = 6.4) (12), the minimum sample size per group was calculated to be 22 cases. Accounting for a 20% potential loss to follow-up, each group required at least 26 participants.

The inclusion criteria were as follows: (1) the infant or young child was in the self-feeding transition period; (2) feeding disorders were found in their feeder's chief complaints and the Montreal Children's Hospital Feeding Scale (MCH-FS) score was >50; (3) the infant or young child had weight loss or no weight gain for at least 1 month; (4) non-organic feeding disorders were diagnosed.

The exclusion criteria were as follows: (1) infant or young child who was born prematurely; (2) infant or young child who had congenital diseases, gastrointestinal diseases, intellectual disability, cerebral palsy, genetic diseases, endocrine or metabolic diseases; or (3) infant or young child who had acute illness in the recent 1 month.

According to the feasibility and effectiveness of the study, the research team formulated the following removal criteria: (1) incomplete data due to poor compliance of feeder; (2) participant declined participation or withdrew from the study; or (3) the number of the shared medical appointment interventions that the participant attended was ≤2 times. In clinical practice, organic and non-organic factors often coexist. For instance, children with a history of allergies are more prone to behavioral food refusal. To avoid over-idealization of study population, this research included infants and young children with histories of allergies, diarrhea, and vomiting, all of whom were in non-acute symptomatic phases.

### 2.3 Shared medical appointment (SMA)

This study adopted multidisciplinary shared medical appointment as a new intervention method. The shared medical appointment is a group visit in which 5–15 patients see a multidisciplinary team of 2 or more healthcare professionals online or offline. A shared medical appointment lasts 90–120 min, and patients need to attended 3–12 shared medical appointments for interventions (13). Compared with traditional intervention methods, shared medical appointments involve individual diagnosis and health care in a group setting as well as group lectures and discussions. This approach can effectively utilize healthcare resources, implement continuous planning, and set clear goals, improving patient experience and self-management of disease (14). The multidisciplinary team in our study consisted of 10 healthcare professionals who were pediatricians, specialist nurses, registered dietitians, psychotherapists, and rehabilitation therapists. Infants and young children in the experimental group needed to attend 3 shared medical appointments, with a 1-month interval between the appointments. Each appointment lasted about 120 min.

### 2.4 Procedures

#### 2.4.1 Intervention

**Figure 1:**
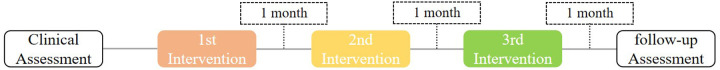
Intervention program procedure.

In this study, the shared medical appointment intervention plan for non-organic feeding disorders in infants and young children during the transition period from exclusive breastfeeding to family foods was drafted by literature review and group discussions. The intervention plan was finally determined after being modified according to the results of expert consultation. The pediatricians provided feeders with health education on the feeding knowledge, instructed parents to follow the feeding principles, and provided some feasible measures to encourage the feeders to respond appropriately and timely to the signals of hunger and fullness in children. They also promoted adaptive feeding practices. The registered dietitians evaluated the variables used in the infant and young child feeding index (such as proportion, frequency, and time of intake of vegetables, fruits, and animal-source foods), provided feeders with age-appropriate meal schedules and information concerning types of food, and organized complementary food preparation salons. The rehabilitation therapists provided instructions for the daily activities of infants and young children. They provided training on the coordination of chewing and swallowing of oral muscles and eating skills. The specialist nurses organized the interventions, collected data, conducted management and follow-up on the study participants, and recorded the behaviors of the feeders. The psychotherapists helped feeders build up feeding confidence base on behavioral and nutritional interventions, thereby reducing the influence of the feeders' negative emotions on feeding interactions. After each intervention, the research team evaluated the intervention effects based on the participants' feedback collected from the WeChat group chat. The feedback was used to improve the feeding situations and help the feeders solve the difficulties they encountered as much as possible. The key to success in this study was that the group communication activities formed a group atmosphere, established a common consciousness, and promoted the change of feeders' behaviors from passive to proactive. This could stimulate the feeders' self-efficacy and achieved the common effect of maintaining intervention measures and extending reasonable feeding intentions. The details of the intervention plan are shown in Table 1. The intervention program procedure are shown in the Figure 1.

**Table 1:** Intervention program outline.

Time	Themes	Details
1st intervention	1. Recognizing infant and young child feeding difficulties.	① Definition/types/causes, etc. ② Near-term and long-term risks. ③ Positive and negative cases of feeding difficulties.
	2. Group psychological intervention.	① Ice-breaking activity & Self-introduction. ② Main feeder self-anxiety subjective scores. ④ Interactive session for feeders: sharing problems and insights with each other to gain emotional commonality and support. ⑤ Psychotherapists use 3 methods to relieve anxiety through scenarios, examples and demonstrations.
2nd intervention	1. Activity guidance.	Gross motor & Fine motor & outdoor activity
	2. Feeding skills and eating behaviors	① How to train children to swallow/chew. ② How to develop children's independent feeding skills. ③ How to develop good eating behaviors.
	3. Complementary feeding	① Principles of complementary feeding. ② Complementary food cooking and preparing. ③ Infants and young children eating health and safety. ④ Meal schedule.
3rd intervention	1. Feeding behaviors	① Methods of introducing new foods. ② Responsive feeding practice. ③ Selection and supplementation of nutrients during the transition period.
	2. Group psychological intervention.	① How to relieving anxiety. ② Resources available (friends, partners, doctors).
	3. Nutrition salons	① Complementary food preparing and matching (Demonstration). ② Recipes.
Follow-up & feedback		① Main feeder supplement preparation and feeding (video). ② Feedback to feeders for results of improvement.

The control group adopted the traditional face-to-face pediatric healthcare visit model. The feeding and nutrition education content was based on the “Chinese Feeding Guidelines for Infants and Young Children Aged 7–24 Months" (15). Participants received a total of 3 outpatient instruction sessions, with a 1-month interval between the appointments. Each appointment lasted about 10∼30 min. The intervention procedures and data collection for the control group were completed concurrently with the intervention group. To avoid contamination, the multidisciplinary team members of the intervention group did not participate in the counseling sessions for the control group.

#### 2.4.2 Clinical assessment

Data concerning physical findings [such as birth weight (kg), birth length (cm), gender, and age (months)], feeding history [such as duration of breastfeeding (months), and age of introducing complementary foods (months)] and medical history of infants and young children, including their basic family information (such as age and education attainment of caregiver, mother's diet during pregnancy, and household income per capita (CNY per month), were collected. The evaluation indicators used in this study were as follows:

(1) Body measurements. Z-scores for weight-for-age (WAZ), Z-scores for height-for-age (HAZ), and Z-scores for head circumference-for-age (HCZ) were measured according to the indicators for the national growth survey of children in the nine cities of China (2005) (16). These Z-scores assessed the weight, height, and head circumference of the infants and young children.
(2) MCH-FS. The MCH-FS was developed by Maria Ramsay, a psychologist from the Montreal Children's Hospital, Canada. It aims to quickly identify the feeding problems in children aged 6 months to 6 years. It contains a total of 14 items. Each item was scored from 1 to 7, and the total raw scores were obtained by summing the entries, with a scale ranging from 14 to 98 (17). In our study, the Chinese version of the MCH-FS, modified by Dai et al. (18), was used to assess the feeding disorders in infants and young children. The raw scores were converted to standardized scores according to the logit transformation method, and the T-scores >50 indicates proximity to the discriminatory cut-off score based on China norms. The scale used in our study contained the following components: parent's self-assessment of child's growth status (item 12), oral motor function (items 7, 8, and 1), parent's feeding behaviors (items 1, 2, 9, 10, 13, and 14), and eating behaviors of child (items 3, 4, 5, and 6). In this study, we will use items 2 and 4 as a proxy for self-efficacy. Studies by Chinese scholars have confirmed that the Chinese version of the MCH-FS has good reliability and stability and has been widely used as a standardized screening tool for feeding disorders in China (19, 20).
(3) Infant and Child Feeding Index (ICFI). The ICFI was first proposed by Ruel and Menon in 2002 (21). Our study adopted the Chinese version of the ICFI for children aged 6–24 months. The Chinese version of the ICFI was developed by Yan et al. (22), and contains 7 components: continued breastfeeding, use of bottles, dietary types, dietary intake frequency in the past 24 h, number of days for complementary feeding in the past week, age of child ingested formula powder for the first time, and age of child ingested other complementary foods for the first time except formula powder. A score ranging from 0 to 2 is assigned to each item. The maximum scale score is 23 (22). The scale score is positively correlated with the feeding status. The higher the scale score, the better the feeding behavior. A low scale score reflected that irrational feeding behaviors exist. This scale can comprehensively assess the feeding status of infants and young children during the transition period from exclusive breastfeeding to family foods. Hence, the scale can effectively evaluate the correlations between feeding practice and feeding outcomes and has predictive value for physical development (23).
(4) Self-Rating Anxiety Scale (SAS). The SAS contains 20 items. A score ranging from 1 to 4 is assigned to each item. Our study referred to the standardized score of the Chinese norm. The Cronbach's alpha coefficient is 0.896 and the content validity is 0.912, indicating good consistency. This scale can assess the subjective feelings of anxiety of feeders and the changes before and after the intervention (24, 25).

### 2.5 Data collection

All participants completed the MCH-FS, ICFI and SAS before the intervention (T1) and 1 month after the intervention (T2). Body measurements of the children were measured before the intervention (T1), 1 month after the intervention (T2), and 3 months after the intervention (T3), respectively. The physical growth indicators were measured by specialist nurses from the research team using the calibrated child height and weight measurement scales and head circumference measuring tape. The feeding questionnaires were filled in by the feeders under the instructions of specialist nurses in accordance with unified standards. The returned questionnaires were checked one by one to ensure their completeness and validity.

### 2.6 Statistical analysis

SPSS Statistics 25.0 was used for data analysis. The *α* level was set as 0.05. A statistically significant difference was identified by *P* < 0.05. The baseline data was described by frequency, constituent ratio, mean ± standard deviation, and M (P25, P75). The inter-group comparison was performed using the *t*-test, Chi-square test, Mann–Whitney *U* test, and Fisher's precision probability test. The physical growth indicators were described with the mean ± standard deviation. The *t*-test, one-way analysis of variance, and repeated measures analysis of variance were used in this study. The MCH-FS, ICFI and SAS scores were described using mean ± standard deviation and compared using the *t*-test. A multivariate regression analyses was employed to account for potential confounding variables that might influence the results.

## 3 Results

### 3.1 Sample characteristics

In the intervention group, 2 cases were removed from the study due to insufficient intervention time, and 1 case was lost to follow-up. In the control group, 1 case was lost to follow-up due to moving home, and 2 cases withdrew from the study because feeders declined the follow-up. Therefore, 46 cases were finally included in this study (23 cases in the intervention group and 23 cases in the control group (37 aged 6–11 and 9 aged 12–24). The average age of the 46 investigated children was 8.7 months. The youngest participant was 6 months old, and the oldest was 17 months old. Of the 46 cases, the average age at which complementary foods were added first time was 6 months, 43.5% did not receive continued breastfeeding, 28.3% had a history of allergy, 54.3% had a history of eczema, 32.6% had a history of constipation, and 69.6% had sleep problems. No statistically significant differences were identified in baseline data between the two groups. Details are presented in Table 2. We conducted further multivariate regression analyses, taking into account the potential influence of certain covariates (caregiver's type/age/education, household income) on the outcomes. The results showed no statistically significant differences after adjusting for these confounding factors.

**Table 2:** Characteristics of children and their households at baseline (*N* = 46).

Characteristic	Intervention (*n* = 23)	Control (*n* = 23)	Statistical values	*P*
Birth weight^a^ (kg)	3.15 (0.57)	3.20 (0.38)	−0.366	0.716
Birth weight^a^ (cm)	49.35 (2.01)	49.39 (2.29)	−0.068	0.946
Gestational age^a^ (weeks)	39.04 (1.24)	39.05 (1.08)	−0.034	0.793
Age^a^ (months)	8.70 (1.22)	8.78 (1.09)	−0.255	0.800
Gender^c^			0.087	0.768
Male	12 (52.2)	11 (47.8)		
Female	11 (47.8)	12 (52.2)		
Breastfeeding duration^a^ (months)	5.09 (3.06)	3.43 (3.07)	1.828	0.074
Age of introducing complementary food^b^ (months)	6.0 (5.0,6.0)	6.0 (5.0,6.0)	−0.064	0.949
Allergy history^c^			0.965	0.326
No	18 (78.3)	15 (65.2)		
Yes	5 (21.7)	8 (34.8)		
Frequent vomiting^d^			–	1.000
No	21 (91.3)	21 (91.3)		
Yes	2 (8.7)	2 (8.7)		
Frequent diarrhoea^d^			–	1.000
No	20 (87.0)	21 (91.3)		
Yes	3 (13.0)	2 (8.7)		
Caregivers^d^			–	0.275
Parents	7 (30.4)	13 (56.5)		
Grandparents	13 (56.5)	9 (39.1)		
Babysitters	2 (8.7)	1 (4.3)		
Others	1 (4.3)	0 (0)		
Caregiver's age^d^ (years)			–	0.178
≤25	0 (0)	1 (4.3)		
26–35	7 (30.4)	9 (39.1)		
36–45	1 (4.3)	4 (17.4)		
>45	15 (65.2)	9 (39.1)		
Education of caregivers^d^			–	1.000
High school or below	10 (43.5)	9 (39.1)		
College	3 (13.0)	4 (17.4)		
Bachelor	9 (39.1)	9 (39.1)		
Master or above	1 (4.3)	1 (4.3)		
Mother's diet during pregnancy^d^			–	0.881
No	16 (69.6)	18 (78.3)		
Poor appetite	4 (17.4)	4 (17.4)		
Picky/parochial Eating	2 (8.7)	1 (4.3)		
Excessive	1 (4.3)	0 (0)		
Mother/Father diet^d^			–	0.267
No	17 (73.9)	21 (91.3)		
Poor appetite	2 (8.7)	2 (8.7)		
Picky/parochial eating	3 (13.0)	0 (0)		
Excessive	1 (4.3)	0 (0)		
Household income per capita^d^ (yuan/month)			–	0.242
≤2,000	0 (0)	0 (0)		
2,001–5,000	3 (13.0)	0(0)		
5,001–10,000	5(21.7)	7(30.4)		
≥10,001	15(65.2)	16(69.6)		

^a^ *M* (SD) = mean(standard deviation), Independent-Sample *t*-test.

^b^ *M* (Q₁, Q₃) = median(1st Quartile, 3st Quartile), Mann–Whitney *t*-test.

^c^ *n* (%) = numbers(percentage), *χ^2^* Chi-square test.

^d^ *n* (%) = numbers (percentage), Fisher's precision probability test.

### 3.2 Body measurements

The results of repeated measures analysis of variance showed that time had an effect on the changes in weight, height, and head circumference in infants and young children in the two groups, as shown in Table 3. The results of the inter-group comparison showed that no statistically significant differences were identified in physical growth indicators between the two groups at different time points (*P* > 0.05). One-way analysis of variance was used for intra-group comparison. The results showed that the Z-scores for the three physical growth indicators in the intervention group after the intervention were higher than those before the intervention (*P* < 0.05); a statistically significant difference was identified in the Z-scores for height-for-age in the control group before and after the intervention (*F* = 5.370, *P* = 0.008); no statistically significant differences were identified in the Z-scores for weight-for-age and head circumference-for-age before and after the intervention (*P* > 0.05), as shown in Table 4. The results of the intra-group pairwise comparison are shown in Table 5.

**Table 3:** Repeated-measures ANOVA for body measurements of infants in the two groups.

Categories	Time effect		Between-subjects effect		Interaction effect	
	*F*	*P*	*F*	*P*	*F*	*P*
WAZ	**7**.**652**	**0**.**001**	0.008	0.931	1.174	0.314
HAZ	**10**.**233**	**<0**.**001**	0.001	0.974	0.063	0.898
HCZ	**7**.**199**	**0**.**002**	1.010	0.320	1.689	0.195

WAZ: Z-scores of weight-for-age.

HAZ: Z-scores of height-for-age.

HCZ: Z-scores of Head Circumference -for-age.

Statistically significant results are highlighted in bold.

**Table 4:** A comparison of body measurements [*M* (SD)]*.*

Categories	Groups	T1	T2	T3	*F*	*P*
WAZ	Intervention	−0.58 (0.95)	−0.56 (1.01)	−0.29 (1.15)	7.460	**0**.**002**
	Control	−0.53 (0.70)	−0.46 (0.64)	−0.37 (0.56)	1.602	0.213
	*t*	−0.213	−0.374	0.304		
	*p*	0.832	0.710	0.763		
HAZ	Intervention	−0.51 (0.85)	−0.41 (0.97)	−0.22 (0.97)	4.997	**0**.**021**
	Control	−0.48 (0.80)	−0.40 (0.71)	−0.24 (0.71)	5.370	**0**.**008**
	*t*	−0.109	−0.043	0.057		
	*p*	0.913	0.966	0.955		
HCZ	Intervention	−0.19 (0.89)	−0.23 (0.82)	0.00 (0.86)	7.520	**0**.**002**
	Control	0.09 (0.80)	0.06 (0.77)	0.15 (0.76)	1.021	0.345
	*t*	−1.097	−1.248	0.495		
	*p*	0.278	0.219	0.550		

T1: before the intervention. T2: 1 month after the intervention. T3: 3 month after the intervention.

Statistically significant results are highlighted in bold.

**Table 5:** Intra-group pairwise comparison of body measurements.

Categories	Groups	Times		D.M.	S.E.	95% CI		*P*
						Upper-bound	Lower-bound	
WAZ	Intervention	T2	T1	−0.023	0.062	−0.153	0.106	0.710
		T3		−0.287	0.096	−0.485	−0.088	**0**.**007**
		T3	T2	−0.263	0.086	−0.441	−0.085	**0**.**006**
	Control	T2	T1	−0.064	0.090	−0.250	0.122	0.485
		T3		−0.153	0.100	−0.360	0.054	0.140
		T3	T2	−0.089	0.064	−0.221	0.043	0.176
HAZ	Intervention	T2	T1	−0.097	0.070	−0.242	0.048	0.178
		T3		−0.280	0.114	−0.516	−0.044	**0**.**022**
		T3	T2	−0.183	0.080	−0.349	−0.016	**0**.**033**
	Control	T2	T1	−0.082	0.078	−0.243	0.079	0.303
		T3		−0.239	0.089	−0.424	−0.054	**0**.**014**
		T3	T2	−0.157	0.050	−0.261	−0.054	**0**.**005**
HCZ	Intervention	T2	T1	0.043	0.053	−0.067	0.152	0.429
		T3		−0.190	0.073	−0.341	−0.040	**0**.**016**
		T3	T2	−0.233	0.065	−0.367	−0.099	**0**.**002**
	Control	T2	T1	0.023	0.063	−0.109	0.155	0.720
		T3		−0.061	0.075	−0.217	0.095	0.426
		T3	T2	−0.084	0.037	−0.161	−0.007	**0**.**034**

DM, deviation mean; SE, standard error; CI, confidence interval.

Statistically significant results are highlighted in bold.

### 3.3 MCH-FS scores

**Figure 2:**
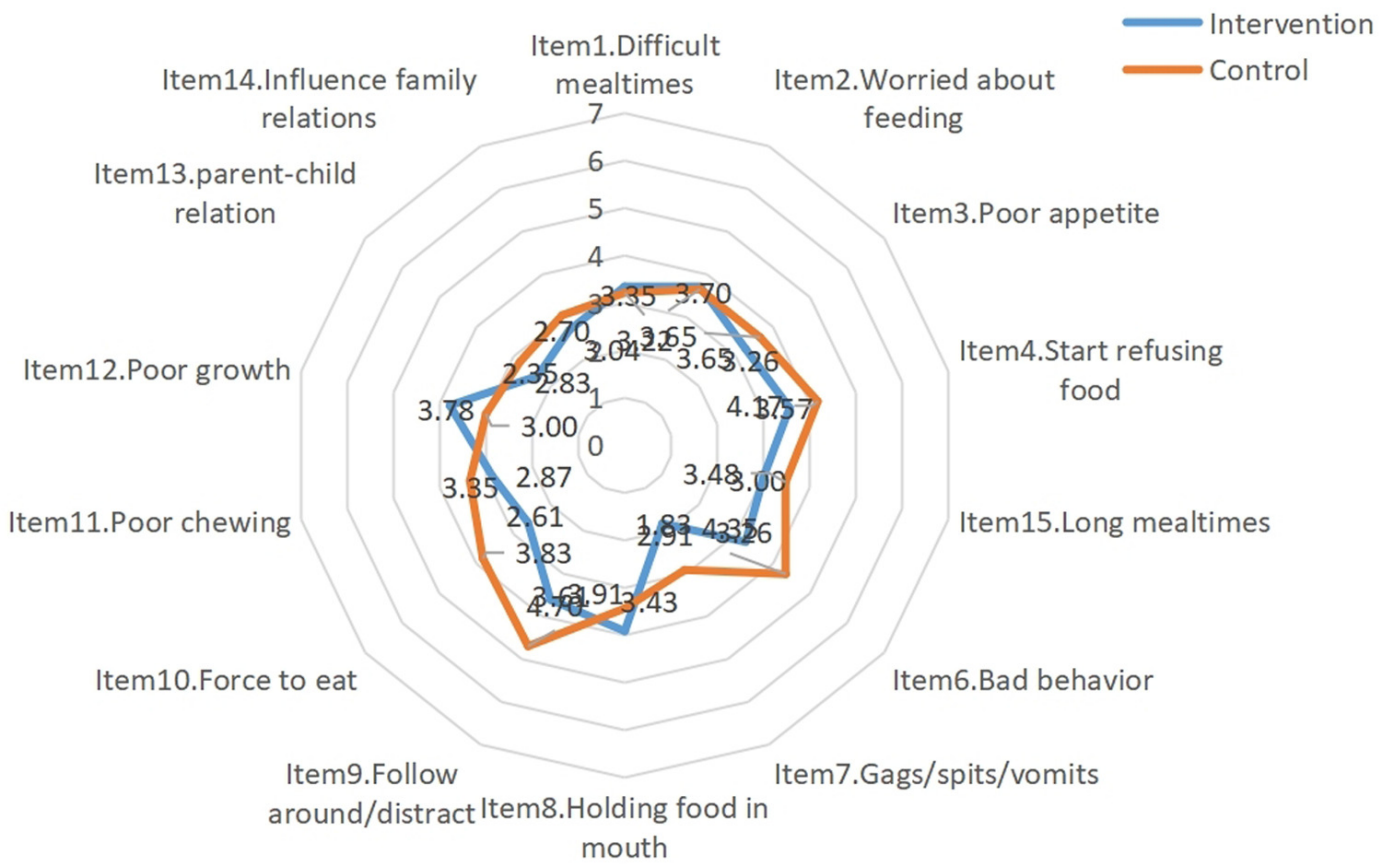
Mean score of the 14 items of the MCH-FS before the intervention.

**Figure 3:**
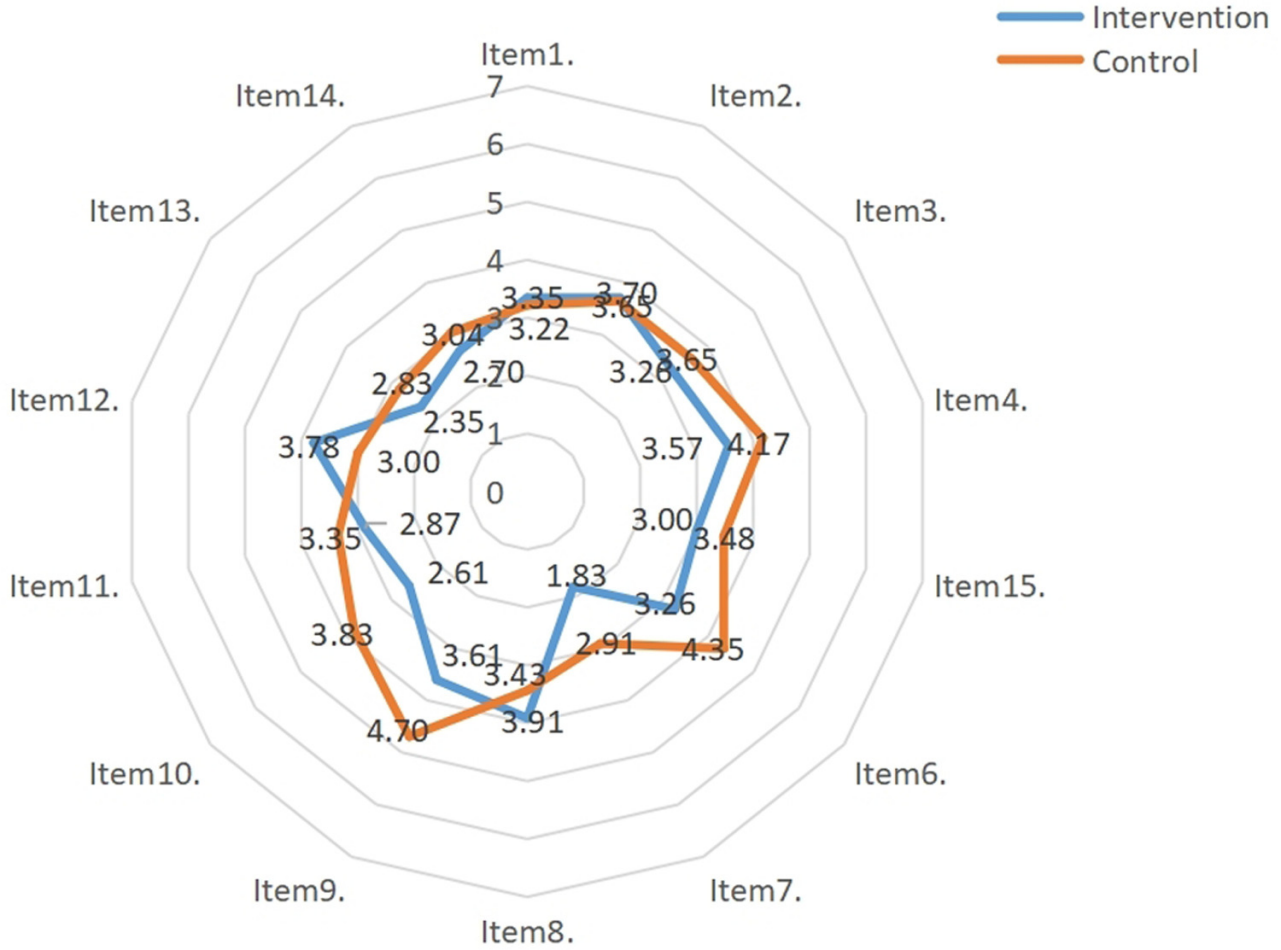
Mean score of the 14 items of the MCH-FS after the intervention.

After the intervention, statistically significant differences were identified in scale scores, feeding behavior scores, and eating behavior scores between the two groups (*t* = −2.073, *P* value for scale scores = 0.046; *t* = −2.106, *P* value for feeding behavior scores = 0.042; *t* = −2.558, *P* value for eating behavior scores = 0.016). The results of the intra-group comparison showed that scale score, feeding behavior score, and eating behavior score in the experimental group after the intervention were significantly lower than those before the intervention (*t* = 3.650, *P* value for scale scores = 0.001; *t* = 4.981, *P* value for feeding behavior scores < 0.001; *t* = 3.283, *P* value for eating behavior scores = 0.003), as shown in Table 6. The mean scores for the MCH-FS items in the two groups before and after the intervention are shown in Figures 2 and 3.

**Table 6:** A comparison of MCH-FS scores [*M* (SD)].

Categories	Groups	T1	T2	*t*	*P*
Growth status	Intervention	3.96 (1.85)	3.78 (1.76)	0.414	0.683
	Control	3.35 (2.06)	3.00 (1.62)	1.017	0.320
	*t*	1.056	1.569		
	*p*	0.297	0.124		
Oral motor	Intervention	8.52 (3.60)	8.61 (2.43)	−0.106	0.916
	Control	8.22 (2.81)	9.70 (2.87)	−1.815	0.083
	*t*	0.319	−1.388		
	*p*	0.751	0.172		
Feeding behavior	Intervention	23.87 (4.85)	18.0 (5.82)	4.981	**<0**.**001**
	Control	22.26 (4.93)	21.26 (3.39)	0.886	0.385
	*t*	1.116	−2.106		
	*p*	0.270	**0.042**		
Eating behavior	Intervention	15.70 (3.10)	13.09 (4.46)	3.283	**0**.**003**
	Control	14.74 (4.01)	15.65 (17.80)	−1.057	0.302
	*t*	0.905	−2.558		
	*p*	0.370	**0.016**		
Total	Intervention	64.83 (7.60)	58.91 (9.21)	3.650	**0**.**001**
	Control	62.39 (7.49)	63.39 (4.75)	−0.596	0.557
	*t*	1.094	−2.073		
	*p*	0.280	**0.046**		

Statistically significant results are highlighted in bold.

### 3.4 ICFI and SAS scores

The results of the inter-group comparisons showed that a statistically significant difference was identified in the ICFI score after the intervention (*t* = 5.814, *P* *<* 0.001); the results of the intra-group comparisons showed that the ICFI score of the experimental group after the intervention was higher than that before the intervention (*t* = −3.689, *P* = 0.001); no statistically significant differences were identified in feeders' SAS scores before and after the intervention. Details are shown in Table 7.

**Table 7:** A comparison of ICFI & SAS scores [*M* (SD)].

Categories	Groups	T1	T2	*t*	*p*
ICFI	Intervention	13.09 (3.77)	16.04 (1.61)	−3.689	**0**.**001**
	Control	13.09 (2.76)	12.65 (2.29)	0.679	0.504
	*t*	0.000	5.814		
	*p*	1.000	**<0.001**		
SAS	Intervention	40.83 (8.90)	40.91 (9.49)	−0.049	0.961
	Control	40.91 (6.35)	39.13 (5.64)	1.411	0.172
	*t*	−0.038	0.775		
	*p*	0.970	0.444		

Statistically significant results are highlighted in bold.

## 4 Discussion

This study found that approximately 43.5% of infants and young children did not receive continued breastfeeding. This shows that early cessation of breastfeeding may be one of the important preconditions for the occurrence of feeding disorders. In addition, this study found that many infants and young children had a history of allergies, eczema, and constipation. The uncomfortable eating experience brought by such problems might be more likely to induce feeding disorders. In our study, 47.8% of the main feeders were grandparents, 52.2% were aged >45 years, and 41.3% possessed a high school diploma or below. This was consistent with the characteristics of the family situations in China. Most of the parents of the surveyed infants and young children are first-generation, only-child Chinese. Both parents of many of the infants and young children included in this study had jobs, so many of the main feeders in this study were grandparents. Some families changed the feeders frequently. Grandparents' feeding concepts might be different with those of other family members. Grandparents might pay insufficient attention to feeding disorders and possess insufficient knowledge of complementary feeding. As such, there were certain misunderstandings in eating behaviors, eating environment, and feeding methods. To address these issues, promoting breastfeeding more vigorously is essential, as it may significantly reduce the occurrence of feeding disorders in infants and young children. It is suggested that more attention should be paid to infants and young children who had a history of allergy, constipation, and eczema and who were fed by grandparents during the transition period from exclusive breastfeeding to family foods. It is recommended that grandparents' feeding knowledge should be improved, and parents are encouraged to be involve in the feeding process so as to form a scientific family feeding mode gradually. This study primarily reflects the feeding characteristics of infants aged 6–12 months. Although older children (13–24 months) were included to enhance universality, the limited sample size in this age group precluded reliable age-stratified analyses. Future multi-center studies targeting different developmental stages are recommended for further validation.

### 4.1 Intervention effects on infants and young children

The results of this study showed that, after the shared medical appointment interventions, the eating behavior score of the infants and young children in the intervention group went down (items 3, 4, and 6), but their ICFI score went up. This indicates that the multidisciplinary shared medical appointment interventions can effectively improve the clinical manifestations of feeding disorders in infants and young children and improve the overall quality of infant and young child feeding. This is consistent with the results shown by Owen et al. (26). Eating behaviors are acquired through continuous learning and practice, and good eating behaviors can meet the nutritional needs of normal growth of infants and young children. The multidisciplinary shared medical appointment interventions applied in this study included health education on feeding knowledge and guided feeders to follow feeding principles (such as reasonably limiting each meal duration to 30 min). Parents were provided with actionable measures (such as sand timer) and recommended to avoid distracting activities during meals (such as watching TV, using mobile phones, and playing with toys). It also provided the feeders with convenient, age-appropriate, and economical recipes and conducted on-site simulation exercises to prepare complementary foods. This is conducive to the daily practice of feeders. Previous studies have confirmed that the severity of feeding disorders is negatively correlated with the Z-scores for weight-for-age, height-for-age, and head circumference-for-age, and improving related symptoms can benefit physical growth (27, 28). The results of our study showed that the Z-scores for physical growth indicators in the experimental group increased after the intervention. This indicates that multidisciplinary shared medical appointment interventions positively affect the physical growth of infants and young children with non-organic feeding disorders during the self-feeding period. With the change of time (except for the similar Z-scores for height-for-age between the two groups), the Z-scores for weight-for-age and head circumference-for-age in the experimental group increased more than the control group, showing an upward trend. This is consistent with the results of Wang et al. (29). The eating behaviors changed, and the types and amount of food intake increased after the interventions for non-organic feeding disorders in the infants and young children during the transition period from exclusive breastfeeding to family foods. Hence, the weight and head circumference increased significantly. Comparably, height increase lagged behind weight increase and are affected by genetic and environmental factors. Our data collection primarily focused on the immediate effects and short-term retention (T1-T2) of behavioral interventions. This approach is theoretically grounded in the understanding that behavior changes always take precedence over improving physical growth indicators, as the latter tend to demonstrate a certain cumulative effect and lagging effect (T3).

### 4.2 Intervention effects on feeders

Feeding behavior refers to a series of specific measures or behaviors taken by the feeders, which may affect children's food choice, intake amount, and eating behaviors. It is an important environmental factor that can be changed and interfered with. During the transition period, the main feeders are in the dominant position in the feeding process. It is necessary and feasible to intervene in feeders' feeding behaviors to change the eating behaviors and nutritional outcomes of infants and young children. There has been several literature on the effects of behavioral interventions in improving feeding problems (30). The results of our study showed that after the main feeders in the experimental group received shared medical appointment interventions, their feeding behavior scores were significantly reduced (items 6, 9, and 10). This was consistent with the results of Sharp et al. (31). Crucially, our data reveal that traditional counseling (control group) failed to induce feeding difficulties changes over time, as evidenced by stable scores in MCH-FS [pre-intervention: 62.39 (7.49); post-intervention: 63.39 (4.75); *P* = 0.557]. This stagnation contrasts sharply with the progressive improvements observed in the intervention group, underscoring the inadequacy of conventional approaches. These findings provide empirical justification for changing conventional health care in children paradigms. In addition, we used items 2 and 4 of the MCH-FS scale as a proxy for self-efficacy. The results showed that caregivers in the intervention group exhibited reduced scores in both “concerns about eating” (Item 2) and “impact of feeding difficulties on family relationships” (Item 4).

The results of our study showed that the SAS scores for feeders in the experimental group did not change significantly after receiving the shared medical appointment interventions, possibly because the shared medical appointment interventions should ultimately have a comprehensive multidisciplinary effect, which may focus on providing feeding knowledge, developing good feeding behaviors of the feeders and establishing a reasonable feeding mode to help feeders build scientific feeding concepts and confidence while reducing parenting stress. The anxiety of feeders can be affected by various factors, such as family environment and the feeders' psychological state. Studies are showing that feeders of infants and young children who have feeding disorders have greater parenting stress and are more likely to suffer from anxiety and depression (32). The effects of the main feeders' emotional responses on feeding interactions and feeding quality should not be ignored and must be further verified.

## 5 Strengths and limitations

This study focuses on infants and young children aged 6–24 months during the transition period from exclusive breastfeeding to family foods. It adopted an innovative intervention mode of shared medical appointments, aiming to improve the feeders' feeding behaviors. The interventions for non-organic feeding disorders in infants and young children during the transition from exclusive breastfeeding to family foods were implemented, and its application effect was preliminarily confirmed, providing a reference for future intervention studies on feeding disorders in infants and young children. This study has some limitations. First, the duration of the study was not long enough to deeply investigate the long-term effect of shared medical appointments and the correlation between intervention duration and intervention effects. Second, due to the limited sample size, the variation differences in food types, food intake frequencies, and food diversity were not presented. This study did not directly demonstrate changes in feeders’ self-efficacy, emotional dynamics and feeding-related stress, subsequent studies could adopt relevant standardized tools for more accurate measurement.We suggest that in future studies, a mixed-methods approach could be used to explore caregiver-child dynamics and the emotional context of feeding in greater depth in conjunction with qualitative interviews. In this study, we included children with food allergy considerations to enhance the representative of our findings, recognizing that such factors may indirectly influence feeding processes by altering gastrointestinal physiology or children's behavior. However, this inclusion may have introduced some heterogeneity, potentially affecting the assessment and intervention of purely non-organic feeding disorders. We emphasize that the compounding effects of organic factors, including allergies, should be thoroughly considered when developing intervention strategies for feeding disorders.

## 6 Conclusions

The shared medical appointment multidisciplinary interventions used in this study could evaluate the non-organic feeding disorders in infants and young children more comprehensively and formulate a professional intervention plan. This can not only give play to the advantages of various academic disciplines, but also realize the comprehensive effects of multidisciplinary interventions. This mode can alleviate the feeding disorders, improve the eating behaviors of infants and young children, improve the ICFI scores, and improve the feeding behaviors of main feeders. It can help form a rational and scientific feeding mode, further promote physical growth, and improve the nutritional status of infants and young children.

## Data Availability

The original contributions presented in the study are included in the article/Supplementary Material, further inquiries can be directed to the corresponding author.
